# Comparative Analysis of Single-Species and Polybacterial Wound Biofilms Using a Quantitative, *In Vivo*, Rabbit Ear Model

**DOI:** 10.1371/journal.pone.0042897

**Published:** 2012-08-08

**Authors:** Akhil K. Seth, Matthew R. Geringer, Seok J. Hong, Kai P. Leung, Robert D. Galiano, Thomas A. Mustoe

**Affiliations:** 1 Division of Plastic Surgery, Feinberg School of Medicine, Northwestern University, Chicago, Illinois, United States of America; 2 Microbiology Branch, US Army Dental and Trauma Research Detachment, Institute of Surgical Research, Fort Sam Houston, San Antonio, Texas, United States of America; University of Iowa Carver College of Medicine, United States of America

## Abstract

**Introduction:**

The recent literature suggests that chronic wound biofilms often consist of multiple bacterial species. However, without appropriate *in vivo*, polybacterial biofilm models, our understanding of these complex infections remains limited. We evaluate and compare the effect of single- and mixed-species biofilm infections on host wound healing dynamics using a quantitative, *in vivo*, rabbit ear model.

**Methods:**

Six-mm dermal punch wounds in New Zealand rabbit ears were inoculated with *Staphylococcus aureus* strain UAMS-1, *Pseudomonas aeruginosa* strain PAO1, or both, totaling 10∧6 colony-forming units/wound. Bacterial proliferation and maintenance *in vivo* were done using procedures from our previously published model. Wounds were harvested for histological measurement of wound healing, viable bacterial counts using selective media, or inflammatory cytokine (IL-1β, TNF-α) expression via quantitative reverse-transcription PCR. Biofilm structure was studied using scanning electron microscopy (SEM). For comparison, biofilm deficient mutant UAMS-929 replaced strain UAMS-1 in some mixed-species infections.

**Results:**

Bacterial counts verified the presence of both strains UAMS-1 and PAO1 in polybacterial wounds. Over time, strain PAO1 became predominant (p<0.001). SEM showed colocalization of both species within an extracellular matrix at multiple time-points. Compared to each monospecies infection, polybacterial biofilms impaired all wound healing parameters (p<0.01), and increased expression of IL-1β and TNF-α (p<0.05). In contrast, mixed-species infections using biofilm-deficient mutant UAMS-929 instead of wild-type strain UAMS-1 showed less wound impairment (p<0.01) with decreased host cytokine expression (*p*<0.01), despite a bacterial burden and distribution comparable to that of mixed-wild-type wounds.

**Conclusions:**

This study reveals that mixed-species biofilms have a greater impact on wound healing dynamics than their monospecies counterparts. The increased virulence of polybacterial biofilm appears dependent on the combined pathogenicity of each species, verified using a mutant strain. These data suggest that individual bacterial species can interact synergistically within a single biofilm structure.

## Introduction

The management and treatment of chronic wounds continues to be a significant burden on the healthcare system [1]–[6]. The importance of bacterial biofilms to the pathogenesis, and subsequent impaired healing, of these wounds has now been validated through a series of *in vitro* and *in vivo* studies [7]–[22]. Defined as a surface-adhered, complex community of aggregated bacteria within an extracellular polymeric substance (EPS), biofilms demonstrate a number of inherent virulence, defense, and survival mechanisms within a host wound environment that differentiate them from traditionally studied, free-floating, ‘planktonic’ bacteria. The biofilm EPS acts a physical barrier, defending against inflammatory cell phagocytosis, while also potentially inhibiting the activation of complement and the penetration of antibiotics [23]–[27]. Other *in vitro* work has revealed that biofilms can shed planktonic bacteria to act as ‘seeds’ for the development of new, remote biofilm populations [8], [9]. Biofilm survival is also enhanced by the maintenance of phenotypically distinct ‘persister’ cells, which provide sustainability and durability in the face of a host immune response [8], [9]. Individual, species-specific virulence mechanisms, such as intricate cell-cell signaling (quorum-sensing) among *P. aeruginosa* cells and the presence biofilm regulatory molecules *sarA*, *agr*, and *cidA* in *S. aureus*, have also been characterized using several *in vitro* and *in vivo* models [28]–[34].

Integral to our growing understanding of chronic wound biofilms have been recent improvements in imaging and molecular sampling techniques over traditional culture-based methods [12], [35], [36]. In particular, these studies have indicated that the bacterial burden of chronic wounds is often underestimated [11], [37]–[40], with the majority of biofilms within these wounds consisting of mixed bacterial species [8], [10], [13]. Predominant bacteria identified have included various anaerobes, *Staphylococcus aureus*, and *Pseudomonas aeruginosa*, with one study finding an average of 5.4 species per wound [41], while others have reported upwards of 106 different possible bacterial genera within human chronic wounds [12]. Consequently, an understanding of biofilm virulence in the context of multiple, interacting bacterial species is critical for placing biofilm research within a clinical framework. This includes delineating the impact of each individual species within a polybacterial setting, and the potential for synergy between different microbes within one wound, which has been previously reported in other settings [42], [43]. Although *in vitro* studies have provided some insight into these polybacterial interactions [44]–[50], the importance of *in vivo* models for understanding bacteria-bacteria interactions in the face of host defenses cannot be overstated. However, with only a limited number of *in vivo* models available, continued research is needed.

Having previously established an *in vivo* model of single-species wound biofilm in the rabbit ear [51]–[53], the goal of this study was to develop and validate a quantitative, *in vivo* model of polybacterial biofilm in order to understand its impact on wound healing dynamics. Using two of the most commonly encountered wound pathogens, *S. aureus* and *P. aeruginosa*, we verified the presence of both species at multiple time-points, resulting in a greater impact on the host inflammatory response and wound healing in comparison to single-species biofilms. We further investigated the contribution of each species to the virulence of a polybacterial wound biofilm, and the potential for synergy between them, using a biofilm-deficient mutant strain of *S. aureus* within the same polybacterial environment, with an expectation that the presence of a mutant would reduce the biofilm’s overall impact on the host. With these findings, we hoped to lay the foundation for identifying mechanisms critical to the establishment, and maintenance, of a polybacterial biofilm phenotype and its impact on wound healing.

## Methods

### Animals

Under an approved protocol by the Animal Care and Use Committee at Northwestern University, adult female New Zealand white rabbits (3–6 months, ∼3 kg) were acclimated to standard housing and fed ad libitum. All animals were housed in individual cages under constant temperature and humidity with a 12-hour light-dark cycle. A total of 45 animals were used for this study.

### Bacterial Strains and Culture

Wild-type and mutant strains of *S. aureus*, and wild-type strain of *P.* aeruginosa, were utilized for wound infection. These included *P. aeruginosa* wild-type strain PAO1, *S. aureus* wild-type strain UAMS-1 and its biofilm-deficient mutant strain UAMS-929. This mutant is deficient in the accessory regulator protein *sarA*, which is known to modulate the expression of enzymes responsible for polysaccharide intercellular adhesin formation. As one of the critical mediators of biofilm formation, the lack of polysaccharide intercellular adhesin seen with UAMS-929 has been shown to reduce its capacity to form biofilm [31], with a resultant increased susceptibility to topical antibiotics *in vitro* [32] and *in vivo* [54]. *S. aureus* and *P. aeruginosa* strains were grown overnight at 37°C on *Staphylococcus* and *Pseudomonas* Isolation Agar (Hardy Diagnostics, Santa Maria, CA), and cultured in tryptic soy (TSB) and Luria (LB) broth, respectively, at 37°C until log-phase was achieved. Bacteria were harvested and washed in phosphate-buffered saline (PBS) three times by centrifugation at 5,000 rpm for 5-minutes at 20°C. An optical density at the 600-nm wavelength (OD_600_) was measured. An OD_600_ equivalent to 10^6^ colony-forming units (CFU)/µL was predetermined empirically for each strain of bacteria used.

### Wound Protocol and Infection Model

Wounding, bacterial infection, and biofilm formation were adapted from principles established in our previously published *in vivo*, wound biofilm model [51]. Rabbits were anesthetized with intramuscular injection of ketamine (22.5 mg/kg) and xylazine (3.5 mg/kg) mixture prior to surgery. Ears were shaved, sterilized with 70% ethanol, and injected intradermally with 1% lidocaine/1∶100,000 epinephrine at the planned wound sites. Six, 6-mm diameter, full-thickness dermal wounds were created on the ventral ear down to perichondrium and dressed with Tegaderm (3 M Health Care, St. Paul, MN), a semi-occlusive transparent film. Individual wounds were inoculated with different combinations of bacteria on postoperative day (POD) 3 as dictated by the experiment being performed. Bacterial solutions were diluted such that each wound was inoculated with a total of 10^6^ CFU of bacteria at a volume of 10-µL. Polybacterial wounds were inoculated with 5-µL of each bacterial species, followed by ‘mixing’ of the two species-solutions *in vivo* with pipette tip. Bacteria were allowed to proliferate *in vivo* under the Tegaderm dressing. Topical antibiotics (*S. aureus* wounds: Mupirocin (2%) ointment [Teva Pharmaceuticals, Sellersville, PA], *P. aeruginosa* wounds: Ciloxan ointment [Ciprofloxacin 0.3%, Alcon, Fort Worth, TX], polybacterial wounds: combination of both antibiotics) were applied POD4 to eliminate free-floating, planktonic-phase bacteria, leaving a predominately biofilm-phase phenotype. To prevent seroma formation and re-growth of planktonic bacteria, thus maintaining a biofilm-dominant infection, an antimicrobial, absorbent dressing containing polyhexamethylene biguanide (Telfa AMD, Tyco Healthcare Group, Mansfield, MA) was applied to biofilm wounds on PODs 5, 6, and then every other day until harvest. All dressings were checked daily throughout the protocol.

### Harvesting of Wounds

After euthanizing the animals by intracardiac euthasol injection, wounds were harvested for various analyses. For viable bacterial count measurements, polybacterial wild-type wounds were harvested at 48-hour intervals from POD6 to POD12, while bacterial counts were measured at POD12 for all other wounds. Scanning electron microscopy (SEM) to visualize the presence, and structure, of polybacterial wild-type biofilm were performed at POD6, the beginning of the biofilm-predominant ‘steady-state’ [51], and on POD12. Histological analyses and reverse transcription quantitative PCR (RT-qPCR) analysis, to measure mRNA levels of inflammatory cytokines, were all performed on wounds harvested at POD12. All wounds were excised using a 10-mm (histology, SEM, viable bacterial counts) or 7-mm (RT-qPCR) biopsy punch (Acuderm inc., Fort Lauderdale, FL).

### Viable Bacterial Count Measurements

The dorsal side of wounds used for bacterial counts were removed to eliminate the inclusion of bacteria outside of the infected wound surface. To recover bacteria, infected wound samples harvested at different time points were placed in tubes pre-filled with homogenizer beads (Roche, Indianapolis, IN). One-mL of PBS was added to the tube and was homogenized for 90-seconds at 5,000 rpm in a MagNA Lyser homogenizer (Roche Diagnostics, Indianapolis, IN), followed by sonication (Microson Ultrasonic Cell Disrupter, Heat Systems-Ultrasonics, Inc, Farmingdale, NY) for 2 minutes at 6–8 watts to disrupt any biofilm present. The resulting solutions were serially diluted and plated on *S. aureus* Isolation Agar (UAMS-1 wounds), *P. aeruginosa* Isolation Agar plates (PAO-1 wounds), or both (polybacterial wounds) and incubated overnight at 37°C. CFUs were determined by standard colony counting method.

### *In vitro* Bacterial Growth Curves

Growth curves for each bacterial species (UAMS-1 and PAO1) and the biofilm-deficient mutant UAMS-929 were created *in vitro*. A single colony of target bacteria was removed from the agar plate containing overnight culture and inoculated into 10-ml of TSB and incubated overnight at 37°C. The following morning, 100-µL were transferred from the overnight growth into 50-ml of fresh TSB. This represented time point ‘zero’. Beginning at time point ‘zero’, a small aliquot was removed from the sample and measured spectrophotometrically at 600-nm. A second small aliquot was taken, serially diluted, and then plated on agar plates in order to obtain bacterial counts. The agar plates were incubated overnight at 37°C and counted the next morning to obtain CFU/ml. This step was repeated every 60-minutes for the duration of the experiment (10-hours for wild-type and mutant *S. aureus* and 18-hours for wild-type *P. aeruginosa*). Upon completion of the experiment, the results were graphed and data analyzed. The lag, log and stationary phases of growth were identified using the graph. The log phase of growth was used to obtain the mean generation time for the target organism. The formula for obtaining the mean generation time was as follows:


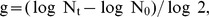


where g =  number of generations in h number of hours, N_t_ is number of bacteria at end of log phase, and N_0_ is number of bacteria at beginning of log phase. Therefore, doubling time in hours was calculated h/g, and in minutes as (h/g) ×60.

### Histological Analysis

Wounds excised for histological analysis were bisected at their largest diameter for H&E staining. Tissues were fixed in formalin, embedded in paraffin, and cut into 4-µm sections. Paraffin was removed with a xylene wash, followed by a standard hematoxylin and eosin staining protocol to prepare samples for analysis under a light microscope. Slides were examined for quantification of epithelial and granulation gaps, and total epithelial and granulation areas, using a digital analysis system (NIS-Elements Basic Research, Nikon Instech Co, Kanagawa, Japan), as previously described [51]. Two blinded, independent observers evaluated all histological sections. The results of both examiners were averaged.

### Scanning Electron Microscopy

To determine biofilm structure, wound samples were fixed in 2.5% glutaraldehyde in 0.1 M phosphate-buffered solution (PBS) (pH 7.2), washed 3× in PBS, and dehydrated through an ethanol series and hexamethyldisilazane. Samples were mounted by double-sided tape to specimen stubs, followed by gold-platinum (50∶50) ion coating (108 Auto Sputter Coater, TedPella, Inc). Wounds for SEM had their dorsal sides removed prior to preparation to allow for better mounting for visualization. Samples were visualized using a Carl Zeiss EVO-40 scanning electron microscope operated at the scanning voltage of 10-kV.

### Total mRNA Extraction and Reverse-Transcription qPCR

Wounds were harvested for mRNA extraction and subsequent cDNA conversion as part of RT-qPCR. The dermal layer on the dorsal side of the ear was removed and the wound bed was punched out and immediately snap-frozen in liquid nitrogen. Wound samples were homogenized using a Mini-bead beater-8 equipment (Biospec Products Inc, Bartlesville, OK) using Zirconia beads (2.0 mm diameter, Biospec Products Inc) in the presence of Trizol Reagent (Sigma-Aldrich, St. Louis, MO). Total RNA was isolated according to the manufacturer’s protocol. Contaminating genomic DNA during RNA preparation was removed using the Turbo DNA-free kit (Ambion, Austin, TX). Five-µg of total RNA was used to prepare cDNA using superscript II (Invitrogen) with 100-ng of random primers (Invitrogen).

For quantitative analysis of the level of mRNAs, RT-qPCR analyses using SYBR green 1 were performed utilizing an ABI prism 7000 sequence detection system (Applied Biosystems, Foster City, CA). PCR primers were designed using the Primer 3 program (http://frodo.wi.mit.edu/). Expression of each gene was normalized to the level of glyceraldehyde 3-phosphate dehydrogenase (Gapdh), the house keeping gene, to get ΔCt. The 2^−ΔΔCt^ method was used to calculate gene expression of IL-1β and TNF-α within the wounds of interest. Expression of genes was detected by PCR with the following oligonucleotides: IL-1β (5′- CCACAGTGGCAATGAAAATG -3′ and 5′- AGAAAGTTCTCAGGCCGTCA -3′, accession number D21835), TNF-α (5′- CCAGATGGTCACCCTCAGAT -3′ and 5′-TGTTCTGAGAGGCGTGATTG-3′, accession number M12845), Gapdh (5′-AGGTCATCCACGACCACTTC-3′ and 5′-GTGAGTTTCCCGTTCAGCTC-3′, accession number NM_001082253).

### Statistical Analysis

Data are presented in graphical form as mean ± standard errors when applicable. Statistical analyses were performed using the Student’s t-test (two-tailed, unpaired) when comparing two study groups, and the Kruskal-Wallis, one-way, analysis of variance (ANOVA) when comparing the means of multiple groups. The level of significance was set at p<0.05.

## Results

**Figure 1:**
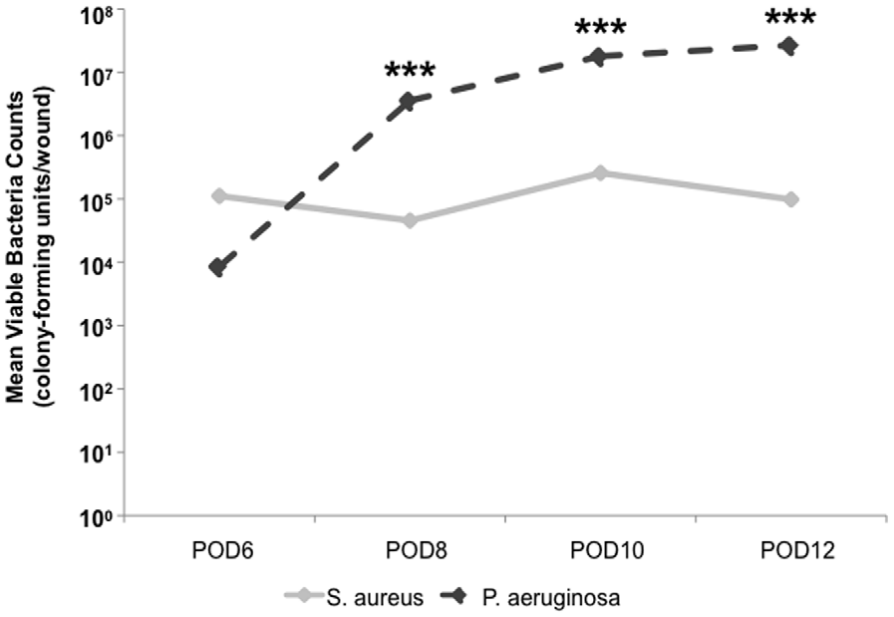
Mean viable bacterial counts from wild-type polybacterial wounds over time. Measurement of viable bacteria from polybacterial wounds demonstrates an increased presence of UAMS-1 relative to PAO1 at POD6. However, by POD8, there is a significant increase in the level of viable PAO1, which is maintained as the predominant bacterial species through POD12. (***p<0.0001) (n = 12 wounds/time point).

**Figure 2:**
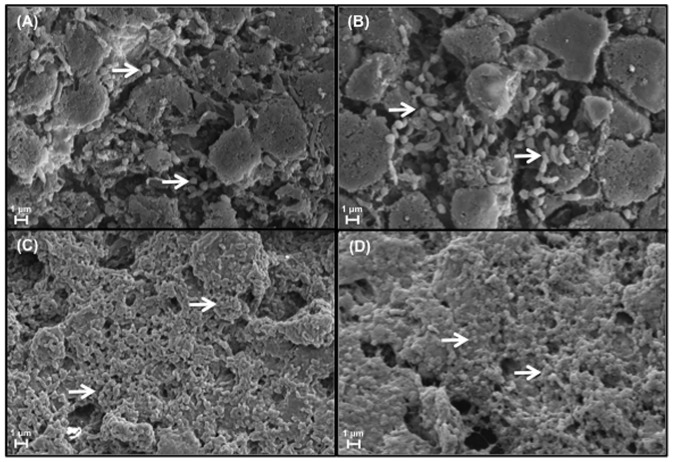
Scanning electron microscopy images of polybacterial wounds at POD6 and POD12. Correlating with bacterial count measurements, wounds at POD6 (A) demonstrate a greater amount of cocci-shaped, *S. aureus* (arrows), while at POD12 (C) a predominance of rod-shaped, *P. aeruginosa* (arrows). However, at both time points there also appears to be spatial colocalization of both species within the wounds (B and D, arrows). (Magnification x25K).

The validation of our *in vivo*, polybacterial model required the verification of viable bacteria, both *S. aureus* and *P. aeruginosa*, within our wounds. Bacterial counts were measured in wounds inoculated with wild-type strains (UAMS-1 and PAO1) of each species every 2-days from POD6 to POD12, revealing the dynamics of the established polybacterial biofilm over time (Figure 1). Following inoculation of equal concentrations of bacteria, UAMS-1 was slightly more predominant than PAO1 at POD6. However, by POD8 and beyond, the number of PAO1 within each wound was significantly higher than UAMS-1 at each measured time point (*p*<0.001). Demonstrated visually, SEM of wounds at POD6 and POD12 revealed a predominance of *S. aureus* and *P. aeruginosa*, respectively (Figures 2A and 2C). However, at both time points, wounds were also found to have areas of co-localization of both species surrounded by an extracellular matrix. (Figures 2B and 2D).

**Figure 3:**
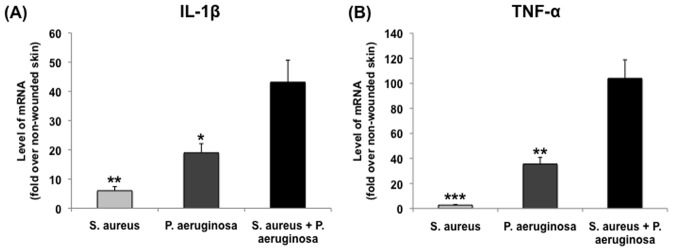
Comparison of inflammatory cytokine mRNA levels between wild-type single-species and polybacterial biofilm wounds. Polybacterial wounds, containing both UAMS-1 and PAO1, demonstrated significantly elevated levels of IL-1β (A) and TNF-α (B) relative to single species wounds. (*p<0.05, **p<0.01, ***p<0.001) (n = 8–10 wounds/group).

**Figure 4:**
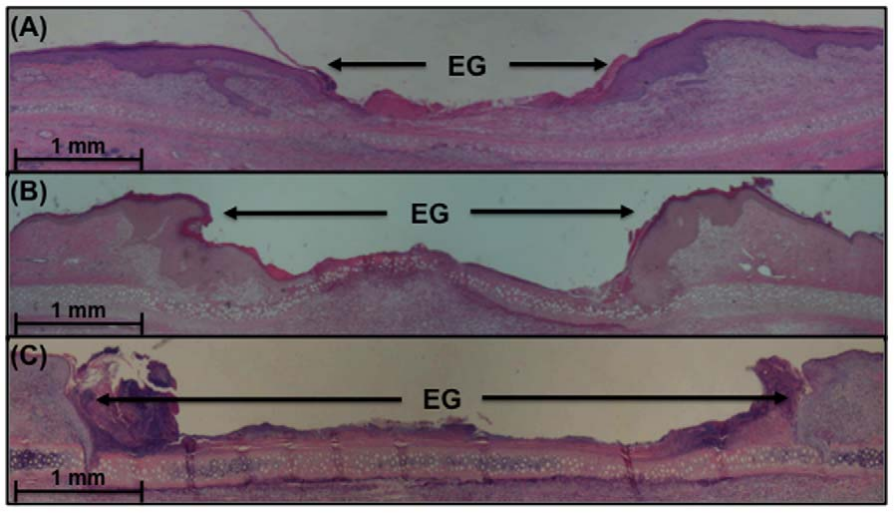
Histological sections from wild-type single species and polybacterial biofilm wounds stained with hematoxylin and eosin. Wounds with *S. aureus* (A) or *P. aeruginosa* (B) demonstrate visually decreased amount of epithelial and granulation tissue relative to wild-type, polybacterial wounds containing both species (C). Note the significant differences in epithelial gap (EG) between polybacterial and single-species wounds. (Magnification x20).

**Figure 5:**
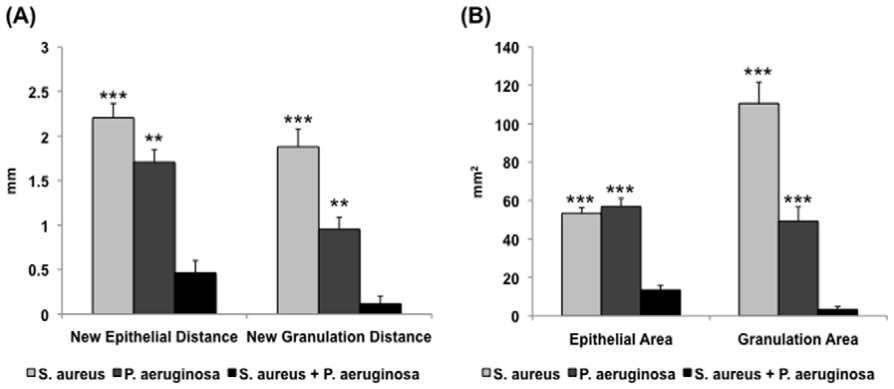
Quantification of histological parameters for wild-type single-species and polybacterial wounds. Measurements of epithelial and granulation gaps (A) and new epithelial and granulation tissue area (B) reveal that polybacterial biofilm significantly impaired wound healing relative to both UAMS-1 and PAO1 alone. (**p<0.01, ***p<0.001) (n = 16–20 wounds/group).

Having established the simultaneous presence of both UAMS-1 and PAO1 within a single wound, we utilized our model to understand the impact of polybacterial biofilms on the host inflammatory response, relative to their corresponding single-species biofilm wounds (Figure 3). The mRNA levels of inflammatory cytokines IL-1β and TNF-α were measured through RT-qPCR for each species, followed by normalization to the level seen in non-wounded skin. For both cytokines, polybacterial biofilm wounds showed higher levels of mRNA expression relative to wounds with only UAMS-1 (IL-1β: *p*<0.01, TNF-α: *p*<0.001) or PAO1 (IL-1β: *p*<0.05, TNF-α: *p*<0.01), indicating that polybacterial biofilm triggered a significantly heightened inflammatory response. Histological analysis of single- and dual-species wounds revealed similar trends among the three groups (Figures 4 and 5). Visual inspection showed that polybacterial biofilms severely limited the formation of new epithelial and granulation tissue as compared to their single-species counterparts (Figure 4). Quantifying these findings across several wounds, polybacterial biofilm was found to significantly affect all measured histological wound healing parameters (Figure 5) (*p*<0.01).

**Figure 6:**
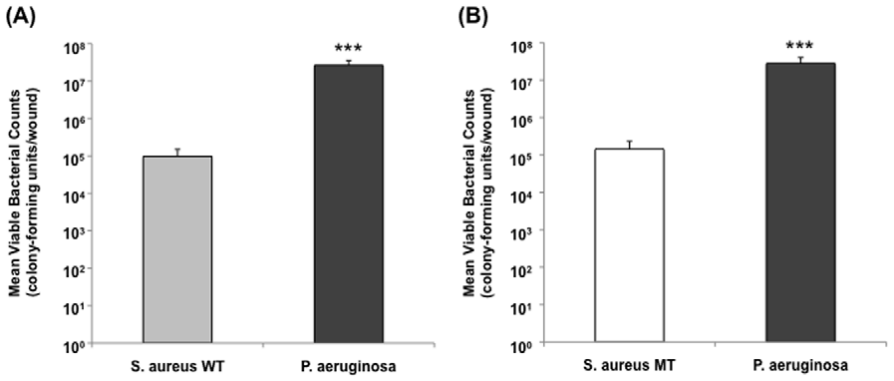
Mean viable bacterial counts for polybacterial wounds containing a wild-type (WT) or mutant (MT) *S. aureus*. Measurement of bacterial counts from polybacterial wounds containing *S. aureus* WT and *P. aeruginosa* (A) demonstrated a similar distribution and total bacterial burden to wounds containing *S. aureus* MT (UAMS-929) and *P. aeruginosa*. In both, there was a significant predominance of *P. aeruginosa* relative to *S. aureus*. (*p*<0.0001) (n = 12 wounds/group).

Given that polybacterial wound biofilm demonstrated a distinct impact on wound healing dynamics relative to single-species biofilms, we aimed to clarify the underlying factors responsible for these differences. With the presence of two different bacteria within a single biofilm, understanding the individual contribution of each species to its overall virulence against the host was of particular importance. Using a biofilm-deficient mutant strain of *S. aureus*, UAMS-929, polybacterial wounds were generated through the combination of wild-type *P. aeruginosa* and UAMS-929, followed by comparison to wounds containing both wild-type strains. Viable bacterial count measurements at POD12 revealed the predominance of *P. aeruginosa* regardless of which type of *S. aureus* was present, wild-type (UAMS-1) or mutant (UAMS-929) (*p*<0.0001) (Figure 6). Furthermore, both polybacterial wounds had a similar distribution of *S. aureus* to *P. aeruginosa* at POD12, regardless of the presence of a mutant strain within the wound. This implied that the *sarA* mutation, responsible for the biofilm-deficiency seen in UAMS-929, did not specifically affect its growth rate, which was confirmed through *in vitro* growth curves generated for each bacterial strain (data not shown).

**Figure 7:**
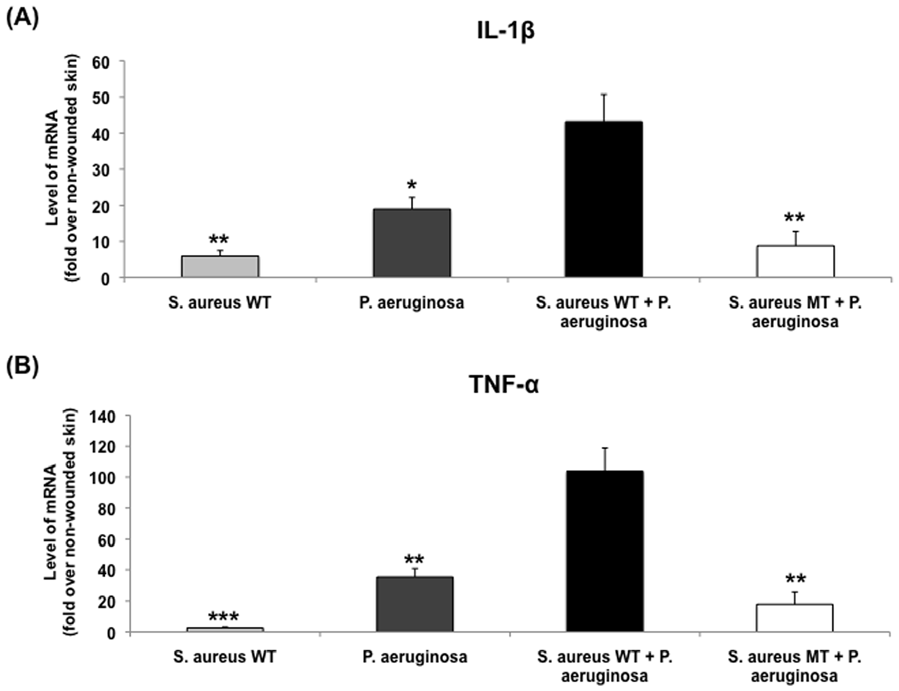
Comparison of inflammatory cytokine mRNA levels between different wound types. Polybacterial wounds containing *S. aureus* mutant (MT) revealed a significantly decreased level of IL-1β (A) and TNF-α (B) expression relative to polybacterial wild-type (WT) wounds containing UAMS-1 and PAO1. The expression level *S. aureus* MT-containing polybacterial wounds were of similar magnitude to single-species WT wounds. (*p<0.05, **p<0.01, ***p<0.001) (n = 8–10 wounds/group).

**Figure 8:**
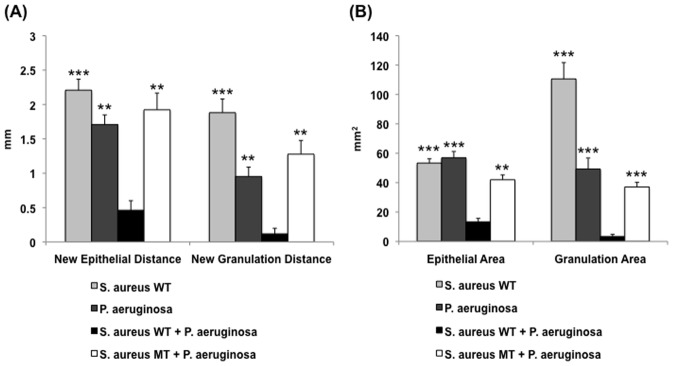
Quantification of histological parameters for different wound types. Polybacterial wounds containing *S. aureus* mutant (MT) UAMS-929 demonstrated decreased impairment in epithelial and granulation gaps (A) and areas (B) relative to polybacterial wounds containing both wild-type (WT) strains. This resulting impairment in wound healing was similar in magnitude to that seen in single-species WT biofilm wounds. (**p<0.01, ***p<0.001) (n = 16–20 wounds/group).

Despite having equivalent growth rates between *S. aureus* mutant and wild-type strains, further analysis of *S. aureus* mutant-containing polybacterial wounds revealed differences in the host inflammatory response that was triggered (Figure 7). The introduction of a UAMS-929 into polybacterial wounds caused a significant reduction in the levels of mRNA for both IL-1β and TNF-α when compared to dual wild-type (UAMS-1+ PAO1) wounds (*p*<0.01). The resultant level of cytokine mRNA expression in the mixed wild-type + mutant wounds was similar to the levels seen in single-species *S. aureus* or *P. aeruginosa* wounds. Correlating with these differences in cytokine expression, similar relationships were found on quantitative histological analysis. Introduction of the *S. aureus* mutant into a polybacterial biofilm reduced its overall impact on wound healing impairment to the level of a single-species bacterial biofilm for all measured histological parameters (Figures 8). Given the known reduction in biofilm formation associated with this *S. aureus* mutant [31], [32], [54], these findings confirmed the importance of each species’ intact biofilm to the polybacterial biofilm’s overall virulence.

## Discussion

The importance of bacterial biofilm to the development, and maintenance, of chronic wounds is now well established within the literature [7]–[22]. With continued improvements in molecular sampling techniques, an increasing amount of literature has reported on the presence of several different bacterial species within these chronic wounds [12], [41]. Therefore, with the majority of research to date focused on biofilms formed by single-species, an *in vivo* understanding of polybacterial biofilm is critical for translating the results of future research to the wounds of human patients. To address this need, we present a polybacterial adaptation of our previously published, *in vivo* wound biofilm model, with the goal of clarifying the dynamics and underlying mechanisms behind polybacterial biofilm.

Previous work with polybacterial biofilm systems has primarily involved *in vitro* systems. Pihl et al [44] studied the interactions between *Staphylococcus epidermidis* and *P. aeruginosa*, demonstrating the *P. aeruginosa* may counteract the colonization of *S. epidermidis* potentially through the extracellular polysaccharides that it produces. Woods et al [50] recently published on their *in vitro* biofilm system, which combined three pathogenic bacteria into one system, finding that the final biofilm structure consisted of distinct layers dominated by one particular species, unlike the colocalization that we noted within our *in vivo* wounds. However, only Dalton et al [55] have previously reported an *in vivo*, polymicrobial biofilm model, but with significant limitations for clinical applicability. The biofilms were established *in vitro*, and then transferred to *in vivo* mouse wounds, which as noted above, would result in a different biofilm organization than the colocalization that we observed. The *in vivo* inoculation of wounds with free-floating, planktonic bacteria in our model allows for the simulation of clinical, wound biofilm pathogenesis. In addition, the mouse model heals almost entirely by contraction, in contrast to human wounds that heal largely by formation of new tissue. They also fail to rigorously evaluate the host response to a polymicrobial biofilm challenge, which we address through an analysis of inflammatory markers. With a superior translatability to clinical wound healing [51], and the sensitivity to quantitatively and qualitatively evaluate multiple endpoints across different bacterial species, we believe our model is distinct in its ability to evaluate the impact of polybacterial biofilm on its host. Furthermore, the model’s flexibility has allowed for the testing of different clinical treatment modalities [52], the introduction of comorbidities (e.g. ischemia [53]) into the wound biofilm system, and the comparison of species-specific virulence between common wound biofilms (submitted for publication). However, with the ultimate goal of better understanding biofilms within human chronic wounds, adapting our *in vivo* model to consistently generate polybacterial biofilm was critical to advancing our work.

Using two bacterial species commonly found within chronic wounds, *S. aureus* and *P. aeruginosa*, we have demonstrated that polybacterial biofilm significantly impairs wound healing relative to its single-species biofilm counterparts, while simultaneously triggering a greater host inflammatory response. This dramatic impairment in wound healing seen with polybacterial biofilm is indicative of a potential synergy or enhancement that occurs between both species within a single wound. The presence of each species within the same wound may lead to molecular and cellular communication, and competition, between each species, leading to an *in vivo* equilibrium based on the individual properties of each bacteria and the wound bed. This is supported by the spatial colocalization, and thus potential interaction, between species seen on SEM. Furthermore, the predominance of *P. aeruginosa* relative to *S. aureus* over time, followed by a plateau in the growth of each, is also representative of this equilibrium. However, as suggested by Dalton et al [55], the end-outcome of this mixed-species milieu may be a joint effect on the dynamics of the wound-healing cascade, resulting in a much lower amount of epithelial and granulation tissue than what is achievable by either single species alone. Similarly, the presence of both species also triggers a substantial increase in the expression of host inflammatory cytokines, indicating that polybacterial wounds are recognized as being phenotypically different by the host. Consequently, given that most chronic wounds contain multiple bacterial species, the overall increase in biofilm virulence seen in our model further underscores the difficulty of eradicating chronic wound biofilm in a clinical setting.

The use of *S. aureus* mutant UAMS-929 within our *in vivo* model has allowed us to speculate on some the mechanisms underlying our findings. In particular, it was important to determine whether our perceived increase in virulence was specifically due to collaboration between multiple, biofilm-forming, bacterial species or simply from an increased total bacterial burden within polybacterial wounds. Interestingly, the biofilm-deficient mutant that we used showed a similar growth rate to its wild-type counterpart *in vitro*, while equilibrating to similar level of bacteria within a polybacterial biofilm *in vivo*. However, despite no change in the distribution and total burden of *S. aureus* and *P. aeruginosa*, polybacterial wounds that contained mutant *S. aureus* demonstrated a distinct reduction in wound impairment and host inflammatory response. As previously mentioned, the *sarA* mutation ultimately modulates the production of polysaccharide intercellular adhesin, critical to many bacterial processes including biofilm extracellular matrix production [31]. This decrease in biofilm durability may potentially reduce the virulence of UAMS-929 relative to wild-type *S. aureus*, as seen through previous work by others [31], [32] and within our *in vivo* model (unpublished data). When used within our polybacterial model, the presence of wild-type *P. aeruginosa* was not able to ‘rescue’ the virulence of a biofilm containing mutant *S. aureus* to the level of a biofilm containing both wild-type strains. These findings suggested that polybacterial biofilm virulence was not dependent on the number of bacteria present, but rather on the ability of each species to effectively maintain, and interact within, a complex biofilm while also expressing virulence factors in the face of host defenses. Without an intact, biofilm-forming mechanism in one of the two species present, the potential synergy between species may be significantly reduced, despite the continued presence of both bacteria within the wound.

Although our findings supplement the available literature on polybacterial biofilm, we acknowledge the limitations of our work. As this study was an initial exploration of *in vivo*, polybacterial biofilm, we did not address the specific cellular and molecular mechanisms responsible for the proposed synergy between species. Future work, using additional mutant strains and sophisticated molecular techniques, will further delineate the importance of different biofilm constituents to the complex interactions within a polybacterial biofilm, but are beyond the scope of these initial observations. Therefore, we believe that our work with UAMS-929 was important in developing a preliminary understanding of the mechanistic principles behind polybacterial biofilm. Our findings represent a growing foundation from which continued research can move forward. Furthermore, our adapted *in vivo,* polybacterial biofilm model continues to provide the distinct advantages associated with our single-species model [51], including multiple, quantitative endpoints with both flexibility in experimental study and reproducibility of results. By now modeling more clinically relevant, polybacterial biofilms, the translatability of our results is further improved, allowing for future mechanistic research and testing of pre-clinical therapeutics.

## References

[1] Fogerty MD, Abumrad NN, Nanney L, Arbogast PG, Poulose B, et al (2008) Risk factors for pressure ulcers in acute care hospitals. Wound Repair Regen 16: 11–18. 18211574 10.1111/j.1524-475X.2007.00327.x

[2] Gordois A, Scuffham P, Shearer A, Oglesby A, Tobian JA (2003) The healthcare costs of diabetic peripheral neuropathy in the US. Diabetes Care 26: 1790–1795. 12766111 10.2337/diacare.26.6.1790

[3] Sen CK, Gordillo GM, Roy S, Kirsner R, Lambert L, et al (2009) Human skin wounds: A major and snowballing threat to public health and economy. Wound Repair Regen 17(6): 763–771. 19903300 10.1111/j.1524-475X.2009.00543.x PMC2810192

[4] Beckrich K, Aronovitch SA (1999) Hospital-acquired pressure ulcers: A comparison of costs in medical vs. surgical patients. Nurs Econ 17: 263–271. 10711175

[5] Ramsey SD, Newton K, Blough D, McCulloch DK, Sandhu N, et al (1999) Patient-level estimates of the cost of complications in diabetes in a managed-care population. Pharmacoeconomics 16: 285–295. 10558040 10.2165/00019053-199916030-00005

[6] Ramsey SD, Newton K, Blough D, McCulloch DK, Sandhu N, et al (1999) Incidence, outcomes, and cost of foot ulcers in patients with diabetes. Diabetes Care 22: 382–387. 10097914 10.2337/diacare.22.3.382

[7] Fleck CA (2006) Fighting infection in chronic wounds. Adv Skin Wound Care 19(4): 184–188. 16641562 10.1097/00129334-200605000-00009

[8] Lindsay D, von Holy A (2006) Bacterial biofilms within the clinical setting: what healthcare professionals should know. J Hosp Infect 64(4): 313–325. 17046102 10.1016/j.jhin.2006.06.028

[9] Parsek MR, Singh PK (2003) Bacterial biofilms: an emerging link to disease pathogenesis. Annu Rev Microbiol 57: 677–701. 14527295 10.1146/annurev.micro.57.030502.090720

[10] Edwards R, Harding KG (2004) Bacteria and wound healing. Curr Opin Infect Dis 17(2): 91–96. 15021046 10.1097/00001432-200404000-00004

[11] James GA, Swogger E, Wolcott R, Pulcini E, Secor P, et al (2008) Biofilms in chronic wounds. Wound Repair Regen 16(1): 37–44. 18086294 10.1111/j.1524-475X.2007.00321.x

[12] Dowd SE, Sun Y, Secor PR, Rhoads DD, Wolcott BM, et al (2008) Survey of bacterial diversity in chronic wounds using pyrosequencing, DGGE, and full ribosome shotgun sequencing. BMC Microbiol 6(8): 43. 10.1186/1471-2180-8-43 PMC2289825 18325110

[13] Gjodsbol K, Christensen JJ, Karlsmark T, Jorgensen B, Jensen AM, et al (2006) Multiple bacterial species reside in chronic wounds: a longitudinal study. Int Wound J 3(3): 225–231. 16984578 10.1111/j.1742-481X.2006.00159.x PMC7951738

[14] Kirker KR, Secor PR, James GA, Fleckman P, Olerud JE, et al (2009) Loss of viability and induction of apoptosis in human keratinocytes exposed to Staphylococcus aureus biofilms in vitro. Wound Repair Regen 17: 690–699. 19671124 10.1111/j.1524-475X.2009.00523.x PMC2749089

[15] Harrison-Balestra C, Cazzaniga AL, Davis SC, Mertz PM (2003) A wound-isolated Pseudomonas aeruginosa grows a biofilm in vitro within 10 hours and is visualized by light microscopy. Dermatol Surg 29(6): 631–635. 12786708 10.1046/j.1524-4725.2003.29146.x

[16] Christensen GD, Simpson WA, Younger JJ, Baddour LM, Barrett FF, et al (1985) Adherence of coagulase-negative staphylococci to plastic tissue culture plates: A quantitative model for the adherence of staphylococci to medical devices. J Clin Microbiol 22(6): 996–1006. 3905855 10.1128/jcm.22.6.996-1006.1985 PMC271866

[17] Sun Y, Dowd SE, Smith E, Rhoads DD, Wolcott RD (2008) In vitro multispecies Lubbock chronic wound biofilm model. Wound Repair Regen 16(6): 805–813. 19128252 10.1111/j.1524-475X.2008.00434.x

[18] Loryman C, Mansbridge J (2008) Inhibition of keratinocyte migration by lipopolysaccharide. Wound Repair Regen 16(1): 45–51. 18211578 10.1111/j.1524-475X.2007.00290.x

[19] Rashid MH, Rumbaugh K, Passador L, Davies DG, Hamood AN, et al (2000) Polyphosphate kinase is essential for biofilm development, quorum sensing, and virulence of Pseudomonas aeruginosa. Proc Natl Acad Sci U S A 97(17): 9636–9641. 10931957 10.1073/pnas.170283397 PMC16917

[20] Davis SC, Ricotti C, Cazzaniga A, Welsh E, Eaglstein WH, et al (2008) Microscopic and physiologic evidence for biofilm-associated wound colonization in vivo. Wound Repair Regen 16(1): 23–29. 18211576 10.1111/j.1524-475X.2007.00303.x

[21] Schierle CF, De la Garza M, Mustoe TA, Galiano RD (2009) Staphylococcal biofilms impair wound healing by delaying reepithelialization in a murine cutaneous wound model. Wound Repair Regen 17(3): 354–359. 19660043 10.1111/j.1524-475X.2009.00489.x

[22] Zhao G, Hochwalt PC, Usui ML, Underwood RA, Singh PK, et al (2010) Delayed wound healing in diabetic (db/db) mice with Pseudomonas aeruginosa biofilm challenge: A model for the study of chronic wounds. Wound Repair Regen 18(5): 467–477. 20731798 10.1111/j.1524-475X.2010.00608.x PMC2939909

[23] Shiau AL, Wu CL (1998) The inhibitory effect of Staphylococcus epidermidis slime on the phagocytosis of murine peritoneal macrophages is interferon independent. Microbiol Immunol 42: 33–40. 9525777 10.1111/j.1348-0421.1998.tb01966.x

[24] Percival SL, Bowler PG (2004) Biofilms and their potential role in wound healing. Wounds 16(7): 234–240.

[25] Johnson GM, Lee DA, Regelmann WE, Gray ED, Peters G, et al (1986) Interference with granulocyte function by Staphylococcus epidermidis slime. Infect Immun 54: 13–20. 3019888 10.1128/iai.54.1.13-20.1986 PMC260109

[26] Lewis K (2001) Riddle of biofilm resistance. Antimicrob Agents Chemother 45: 999–1007. 11257008 10.1128/AAC.45.4.999-1007.2001 PMC90417

[27] Shigeta M, Tanaka G, Komatsuzawa H, Sugai M, Suginaka H, et al (1997) Permeation of antimicrobial agents through Pseudomonas aeruginosa biofilms: A simple method. Chemotherapy 43: 340–345. 9309367 10.1159/000239587

[28] Davies DG, Parsek MR, Pearson JP, Iglewski BH, Costerton JW, et al (1998) The involvement of cell-to-cell signals in the development of a bacterial biofilm. Science 280: 295–298. 9535661 10.1126/science.280.5361.295

[29] Nakagami G, Morohoshi T, Ikeda T, Ohta Y, Sagara H, et al (2011) Contribution of quorum sensing to the virulence of Pseudomonas aeruginosa in pressure ulcer infection in rats. Wound Repair Regen 19: 214–222. 21362089 10.1111/j.1524-475X.2010.00653.x

[30] Nakagami G, Sanada H, Sugama J, Morohoshi T, Ikeda T, et al (2008) Detection of Pseudomonas aeruginosa quorum sensing signals in an infected ischemic wound: An experimental study in rats. Wound Repair Regen 16: 30–36. 18211577 10.1111/j.1524-475X.2007.00329.x

[31] Beenken KE, Blevins JS, Smeltzer MS (2003) Mutation of sarA in Staphylococcus aureus limits biofilm formation. Infect Immun 71(7): 4206–4211. 12819120 10.1128/IAI.71.7.4206-4211.2003 PMC161964

[32] Weiss EC, Spencer HJ, Daily SJ, Weiss BD, Smeltzer MS (2009) Impact of sarA on antibiotic susceptibility of Staphylococcus aureus in a catheter-associated in vitro model of biofilm formation. Antimicrob Agents Chemother 53(6): 2475–2482. 19289527 10.1128/AAC.01432-08 PMC2687244

[33] Yarwood JM, Bartels DJ, Volper EM, Greenberg EP (2004) Quorum sensing in Staphylococcus aureus biofilms. J Bacteriol 186(6): 1838–1850. 14996815 10.1128/JB.186.6.1838-1850.2004 PMC355980

[34] Rice KC, Mann EE, Endres JL, Weiss EC, Cassat JE, et al (2007) The cidA murein hydrolase regulator contributes to DNA release and biofilm development in Staphylococcus aureus. Proc Natl Acad Sci U S A 104(19): 8113–8118. 17452642 10.1073/pnas.0610226104 PMC1876580

[35] McGuckin M, Goldman R, Bolton L, Salcido R (2003) The clinical relevance of microbiology in acute and chronic wounds. Adv Skin Wound Care 16: 12–23. 12582302 10.1097/00129334-200301000-00011

[36] Thomson PD (2000) Immunology, microbiology, and the recalcitrant wound. Ostomy Wound Manage 46: 77S–82S. 10732642

[37] Burmolle M, Thomsen TR, Fazli M, Dige I, Christensen L, et al (2010) Biofilms in chronic infections - a matter of opportunity - monospecies biofilms in multispecies infections. FEMS Immunol Med Microbiol 59(3): 324–336. 20602635 10.1111/j.1574-695X.2010.00714.x

[38] Hill KE, Davies CE, Wilson MJ, Stephens P, Harding KG, et al (2003) Molecular analysis of the microflora in chronic venous leg ulceration. J Med Microbiol 52: 365–369. 12676877 10.1099/jmm.0.05030-0

[39] Davies CE, Hill KE, Wilson MJ, Stephens P, Hill CM, et al (2004) Use of 16S ribosomal DNA PCR and denaturing gradient gel electrophoresis for analysis of the microfloras of healing and nonhealing chronic venous leg ulcers. J Clin Microbiol 42: 3549–3557. 15297496 10.1128/JCM.42.8.3549-3557.2004 PMC497624

[40] Andersen A, Hill KE, Stephens P, Thomas DW, Jorgensen B, et al (2007) Bacterial profiling using skin grafting, standard culture and molecular bacteriological methods. J Wound Care 16: 171–175. 17444383 10.12968/jowc.2007.16.4.27025

[41] Thomsen TR, Aasholm MS, Rudkjobing VB, Saunders AM, Bjarnsholt T, et al (2010) The bacteriology of chronic venous leg ulcer examined by culture-independent molecular methods. Wound Repair Regen 18: 38–49. 20082680 10.1111/j.1524-475X.2009.00561.x

[42] Mastropaolo MD, Evans NP, Byrnes MK, Stevens AM, Robertson JL, et al (2005) Synergy in polymicrobial infections in a mouse model of type 2 diabetes. Infect Immun 73: 6055–6063. 16113326 10.1128/IAI.73.9.6055-6063.2005 PMC1231087

[43] Hendricks KJ, Burd TA, Anglen JO, Simpson AW, Christensen GD, et al (2001) Synergy between Staphylococcus aureus and Pseudomonas aeruginosa in a rat model of complex orthopaedic wounds. J Bone Joint Surg Am 83-A: 855–861. 11407793 10.2106/00004623-200106000-00006

[44] Pihl M, Chavez de Paz LE, Schmidtchen A, Svensater G, Davies JR (2010) Effects of clinical isolates of Pseudomonas aeruginosa on Staphylococcal epidermidis biofilm formation. FEMS Immunol Med Microbiol 59: 504–512. 20579097 10.1111/j.1574-695X.2010.00707.x

[45] Hill KE, Malic S, McKee R, Rennison T, Harding KG, et al (2010) An in vitro model of chronic wound biofilms to test wound dressings and assess antimicrobial susceptibilities. J Antimicrob Chemother 65(6): 1195–1206. 20378671 10.1093/jac/dkq105

[46] Standar K, Kreikemeyer B, Redanz S, Munter WL, Laue M, et al (2010) Setup of an in vitro test system for basic studies on biofilm behavior of mixed-species cultures with dental and periodontal pathogens. PLoS One 5(10): 131–135. 10.1371/journal.pone.0013135 PMC2948514 20957048

[47] Ma H, Bryers JD (2010) Non-invasive method to quantify local bacterial concentrations in a mixed culture biofilm. J Ind Microbiol Biotechnol 37(10): 1081–1089. 20552252 10.1007/s10295-010-0756-z PMC3070421

[48] Sillankorva S, Neubauer P, Azeredo J (2010) Phage control of dual species biofilms of Pseudomonas fluorescens and Staphylococcus lentus. Biofouling 26(5): 567–575. 20544433 10.1080/08927014.2010.494251

[49] Stubblefield BA, Howery KE, Islam BN, Santiago AJ, Cardenas WE, et al (2010) Constructing multispecies biofilms with defined compositions by sequential deposition of bacteria. Appl Microbiol Biotechnol 86(6): 1941–1946. 20180119 10.1007/s00253-010-2473-y

[50] Woods J, Boegli L, Kirker KR, Agostinho AM, Durch AM, et al (2012) Development and application of a polymicrobial, in vitro, wound biofilm model. J Appl Microbiol 112(5): 998–1006. 22353049 10.1111/j.1365-2672.2012.05264.x PMC3324638

[51] Gurjala AN, Geringer MR, Seth AK, Hong SJ, Smeltzer MS, et al (2011) Development of a novel, highly quantitative *in vivo* model for the study of biofilm-impaired cutaneous wound healing. Wound Repair Regen 19(3): 400–410. 21518094 10.1111/j.1524-475X.2011.00690.x

[52] Seth AK, Geringer MR, Gurjala AN, Hong SJ, Galiano RD, et al (2012) Treatment of *Pseudomonas aeruginosa* biofilm-infected wounds with clinical wound care strategies: A quantitative study using an *in vivo* rabbit ear model. Plast Reconstr Surg 129(2): 354–361. 22286442 10.1097/PRS.0b013e31823aeb3b

[53] Seth AK, Geringer MR, Gurjala AN, Abercrombie JA, Chen P, et al (2012) Understanding the host inflammatory response to wound infection: An in vivo study of Klebsiella pneumoniae in a rabbit ear wound model. Wound Repair Regen 20(2): 214–225. 22332606 10.1111/j.1524-475X.2012.00764.x

[54] Weiss EC, Zielinska A, Beenken KE, Spencer HJ, Daily SJ, et al (2009) Impact of sarA on daptomycin susceptibility of *Staphylococcus aureus* biofilms *in vivo* . Antimicrob Agents Chemother 53(10): 4096–4102. 19651914 10.1128/AAC.00484-09 PMC2764180

[55] Dalton T, Dowd SE, Wolcott RD, Sun Y, Watters C, et al (2011) An *in vivo* polymicrobial biofilm wound infection model to study interspecies interactions. PLoS One 6(11): e27317 (1–10). 22076151 10.1371/journal.pone.0027317 PMC3208625

